# Longitudinal analysis of health outcomes after exposure to toxics, Willits California, 1991–2012: application of the cohort-period (cross-sequential) design

**DOI:** 10.1186/1476-069X-13-88

**Published:** 2014-10-24

**Authors:** Linda L Remy, Ted Clay

**Affiliations:** Family Health Outcomes Project, Family and Community Medicine, School of Medicine, University of California San Francisco, 500 Parnassus Ave. Room MU-337, San Francisco, CA 94143-0900 USA

**Keywords:** Cross-sequential, Cohort-by-period, Longitudinal, Population health, Data linkage, Non-occupational exposure, Hexavalent chromium (Cr6)

## Abstract

**Background:**

About 1963, a factory in Willits, Mendocino County (County), California added chrome plating to the manufacture of steel products. After years of residents reporting high illness rates, the State undertook a series of investigations. They found exposures had been high and warranted further research into possible health effects. Applying the seldom-used cross-sequential design, we tested if Willits had an excess rate of adverse health conditions, compared to people of the same sex and cohort living in the rest of county (ROC). This is the first report on long-term health outcomes for Willits.

**Methods:**

Hospital discharge data for 1991–2012 were searched to find admissions for people born between 1940 and 1989 who ever gave the County as their residence. Diagnoses and procedures were classified to Level 1 (body systems) of the Multi-level Clinical Classification Software (CCS). We analyzed 796,917 diagnoses and 289,980 procedures found on 117,799 admissions of 43,234 patients who lived in the County at some time between 1991 and 2012. Of these, 7,564 lived in Willits. We summarized data to the person-level then the group level over cohort-period (cross-sequential) to control the age by time relationship, then calculated incidence rates, relative risk, and excess case statistics, each with confidence limits. A secondary analysis focused on whether Willits differed markedly from the rest of County (ROC). Specifically, other than the presence of the Plant, did Willits differ so much that those differences could plausibly explain outcome differences?

**Results:**

Illness was excessive in the exposed group (Willits) compared to the unexposed (ROC). Overall number of excess cases attributable to living in Willits was estimated: Men, 301 (95% confidence limit (CL) 200–398), women: 696 (CL 569–820).

**Conclusions:**

This study demonstrates the strength of the cross-sequential design. Willits and ROC had comparable disadvantages relative to the State. Yet, when stratified by cohort, Willits had more illness per population. Little is known about the health effect of chemicals used at Willits on a non-occupationally exposed population. We recommend a follow-up study to evaluate the long-term health of people who lived in Willits during childhood and the reproductive age.

**Electronic supplementary material:**

The online version of this article (doi:10.1186/1476-069X-13-88) contains supplementary material, which is available to authorized users.

## Background

### History

About 1950, a company named Abex/Remco Hydraulics (Factory) began to expose air, soil, and water in Willits, Mendocino County (County), California to a variety of toxics (cadmium, nickel, zinc, lead, diesel, 111-Trichloroethane, volatile organic compounds such as trichloroethylene, and others). These were by-products of the Factory, which added hexavalent chromium (Cr6) for chrome plating in the early 1960s and later the manufacture of military components. Some neighborhoods in this small community were more exposed by proximity. A school was (and still is) across the street [1].

In its 55-year history, the Factory changed ownership several times and became the town’s largest employer. It closed in 1995, declaring bankruptcy after years of regulatory investigations [2].

In June 2000, the federal Environmental Protection Agency requested the California Department of Health Services (CDHS) to evaluate the potential health effect on the surrounding community. The CDHS Environmental Health Investigations Branch prepared a series of Public Health Assessments under a cooperative agreement with the Agency for Toxic Substance and Disease Registry (ATSDR). These included studies of Cr6 [1], 111-Trichloroethane [3], trichloroethylene [4] and other toxics, evaluating exposure routes and related matters [5–7]. Investigators reported an increased risk of cancer and non-cancer health outcomes among selected populations exposed to Factory emissions, and ordered soil and groundwater remediation to protect public health [7].

An expert panel convened in 2006 to assess the feasibility of medical monitoring. The panel reported that exposure-related health outcomes probably already had occurred, but it would be difficult to identify and track those exposed since many had moved [8], providing the impetus for this study. Could we use longitudinal health data to identify people who had lived in the County and from this assess health outcomes for Willits? We began to track County residents in 2007, using data from 1991 forward.

### Health effects

Most scientific knowledge about the health effects of toxics comes from studies of animals or adult men who worked in facilities processing toxics [1]. Research supports that toxics used at the Factory affect multiple body systems depending on exposure route [4, 9]. For healthy workers, adverse health conditions increase with dose [1]. However, not all workers experience these and some not even as concentrations increase ten or hundredfold. Genetics and lifestyle may be involved. Some people may be more susceptible and others resistant to the same exposure.

Few non-occupational health studies exist, and little is known about long-term effects of childhood or reproductive period exposures [1, 6]. Willits investigators recognized that infants and children may be more sensitive than adults to environmental exposures, characterized risks to children in the Willits area, and felt information was needed on people who were children at the time of exposure.

Research on the long-term effect of exposure to environmental risks is increasingly well developed [10–13]. However, the literature on the impact of the type of stressors communities affected by toxic contamination experience is sparse: “the stressor consisted of a series of events over months and years, starting with the first reports of chemical contamination, and continuing through the responses of governmental agencies, different investigations, relocation and its aftermath [14]”. To date, identified effects of stress in communities near hazardous sites include cardiovascular disease [14, 15], demoralization [16, 17], and mental health problems [18–20] including increased substance abuse [21, 22].

### Conceptual approach

**Figure 1:**
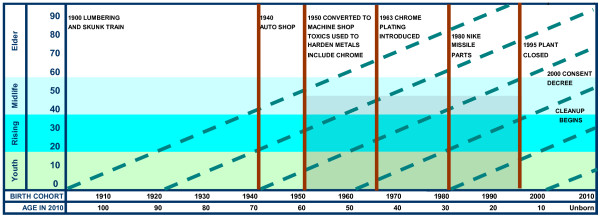
**Time, place, events, and age location in Willits history.**

Strauss and Howe were among the first to define, locate, and name the sequence of American generations and describe how major events of each generation’s time shape them [23]. We adapted their timeline model to visualize longitudinal relationships between birth cohorts, developmental stage (age), and life events. Figure 1 shows relationships between period (horizontal bar), age and developmental stage (vertical bar), and generation (diagonal bar). The vertical bar shows age stages defined by Strauss and Howe: Youth, Rising Adult, Midlife Adult, and Elder. Diagonal bars show each generation’s trajectory across development, period, and events. The first horizontal bar at the bottom shows successive 10-year periods when people were born. The age bar shows how old someone would be at period end, relative to 2010. For example, someone born in 1960 surviving to 2010 would be age 50, and a potential bearer of long-term effects of particular events [24].Vertical bars show key events in Factory history. This was an enduring industrial presence, with gradual changes in potential effect. Figure 1 suggests that generations born 1920 or later were exposed. We are most concerned about cohorts born 1950 and later, particularly people in Willits from 1963 forward when the Factory added chrome plating. As shown by the greyed area, this covers childhood and the reproductive period. By 2012, many are entering elderhood where high profile diseases such as cancer begin to manifest. Given the history of changing exposure, the goal of this study is to assess if community health was affected, and if risk was consistent across cohorts.

Many public health surveillance studies of adverse community effects typically calculate rates at various periods using the standard age by period (time-sequential) design. Willits was no exception: one study examined lung cancer cases [1] and another, cancer deaths [25]. As with many other surveillance reports, both failed to find a significant relationship. The events studied are rare, the community is small, and the population was relatively young, making it unlikely to find an effect, which may be the goal if the intent is to calm community fears or minimize financial liability.

However, another reason findings did not reach statistical significance may be that the researchers examined age effects instead of cohort effects. As Hagenaars stated, “The absence of age-period interactions does not exclude the existence of very strong (linear) cohort effects. . . . Given the relationship between age, cohort, and period, cohort can be omitted only if it is unrelated to the dependent variable ([25], pp. 328,329).” Before employing the standard design, Hagenaars advises researchers to reject the possibility of a cohort effect, which Figure 1 suggests.

## Methods

### Overall design

The County was subdivided into Willits and the rest of the County (ROC). The primary analysis compared health condition rates for residents of these areas from 1991–2012. The numerator of each rate is a count of people born between 1940 and 1989, hospitalized at least once with a given health condition. The denominator is an estimate of the number of person-years County residents in those cohorts could have been hospitalized. Comparisons were within sex and birth cohort [25]. Secondary descriptive analyses assessed comparability of the areas in other ways.

### Geographic descriptors

The term “County” describes the large, rural, and sparsely populated area of Mendocino County. “Willits” (exposed) describes residents of ZIP-code 95490, which the County uses to report public health statistics [26]. Willits population data was based on the same Census ZIP-Code Tabulation Area [27–29]. Both discharge and Census data can identify Willits residents for classifying admissions and population. The comparison is residents in the rest of the County (ROC) (unexposed).

The 2010 Census reported the following about these communities: County, population 87,841, area 3,506 square miles; Willits ZIP-code 95490, population 13,264, area 392 square miles; Willits proper, population 4,888, area 2.8 square miles. The ZIP-code area enclosing Willits proper has dispersed housing located in redwood wilderness, agricultural lands, and small rancherias [30]. The only grocery is by the Factory site.

### Patient discharge data

Our group has confidential hospital patient discharge data (PDD) from California’s Office of Statewide Health Planning and Development for the years 1983–2012. These files were prepared previously for longitudinal research, using methods described elsewhere [31]. We used PDD for the period 1991 to 2012. Data before 1991 lack the Social Security Number (SSN, encrypted to protect confidentiality) to uniquely identify people. Variables used include the patient’s SSN, birthdate, sex, ZIP-code, county of residence and admission, expected source of payment, and principal and up to 24 secondary diagnoses and up to 20 secondary procedures classified based on the International Classification of Diseases, 9th Revision.

### Defining a person

For this study, we define a person as the combination of SSN, sex, and year of birth. Sex was used to identify a person because never-employed spouses can use their partner’s SSN. For patients age 18–64, 97% of Willits admissions had SSN; for ROC, 95%; for California as a whole, 89%. Statewide, SSN was present for virtually all discharges age 65 and older. Thus, we had a high likelihood of identifying adults no longer living in the County if admitted at least once while living there. In a state like California, with a large immigrant population, many people lack a valid SSN. Studies either omit records lacking SSN or use “soft linkage” [32–34]. We decided against soft linkage because of the high percent of patients with SSN. Records lacking SSN and SSN with more than one birth year were excluded.

### Classifying health conditions

To identify health conditions, we used Level 1 of the Multi-level Clinical Classification Software (CCS) developed by the federal Agency for Healthcare Research and Quality (AHRQ) [35]. The CCS clusters diagnoses and procedures into a manageable number of clinically meaningful categories. Level 1 groups by body system and is ideal for evaluating large aggregations of conditions. Following methods used by the Healthcare Cost and Utilization Project [36], measures were identified by searching over all available fields.

We do not report conditions originating during pregnancy and puerperium, because other designs are more appropriate. People born after 1989 were excluded because the methodology is not appropriate for children, who predominantly lack SSN.

### Population estimates

Annual population estimates with detailed age and sex are not available longitudinally at the sub-county level. We tested county-level denominators produced by the State of California [37–39] and Federal government [40–42]. In the end, we used Federal estimates and tied Federal county numbers to Census ZIP-level estimates. For Willits population, we used ZIP-code 95490 from the 1990, 2000, and 2010 censuses [43], extrapolated proportionately over the years, then subtracted the result from the Federal county total to estimate ROC population.

### Birth cohort

Exact linear relationships exist among age, period, and cohort. If two are known, the third can be calculated [24, 25]. A focus on cohort disentangles these confounding effects and is particularly well suited for exploratory studies. It provides a framework to interpret data as an interaction between age and period, namely, the effect of aging during particular historical events.

We calculated cohort as the decade of birth year. In this study, the terms cohort, birth cohort, and birth-year cohort refer to a 10-year period. People born in the same 10-year period belong to the same cohort. Someone born between 1960 and 1969 would be in the 1960 cohort. In the population data, we subtracted age from year. Five-year cohorts produced a small numbers problem.

Stratifying data as we did is known as the cohort-period (cross-sequential) design [25]. We are summing health history for multiple cohorts (1940–1980) over time (and events) within the diagonal lines in Figure 1 for the period 1991 and 2012. The strength of the cross-sequential design is that it untangles the relationship between age and time. This stratification is well suited for longitudinal research focusing on group versus individual outcomes. By contrast, the standard age by period (time-sequential) design monitors public health indicators such as birth or death rates, as represented by vertical bars in Figure 1.

### Analysis files and statistical tests

This is an ecological study. Such studies examine group outcomes, generate hypotheses about the role of environmental factors in individual health, and counterbalance studies emphasizing individual risk [44]. We tested if Willits had different outcomes than ROC, as measured by admissions with one or more mutually exclusive diagnoses or procedures grouped to the Level 1 CCS body system.

We began by extracting discharges with SSN whose birth year was between 1940 and 1989, hospitalized while living in the County. We summarized these to the person-level, then returned to the PDD and found all records after their first admission as a resident. We flagged each time a given condition or procedure was found, and again summarized to the person-level. A person could have 1 to N admissions, 1 to N conditions and 0 to N procedures. Except for number of admissions and days, this analysis used “Any” counts: 1 if found at least once and 0 if never found. Next, we summarized the person file by sex, cohort, and residence, counting number of people, total admissions and days, and affected body systems. Finally, we merged the summary PDD file with the population file, divided summarized PDD counts by population counts, and rescaled the resulting rate per 10,000 person years.

We used Fisher’s Exact Test to test for differences between Willits and ROC within sex and cohort, and calculated Mantel-Haenzel relative risk (RR) and excess cases (EC) statistics including lower (LCL) and upper (UCL) confidence limits (CL). EC are calculated from the RR estimate as follows: EC = number of Willits cases * (1 – 1/RR). RR, LCL and UCL are from the “Cohort Study” row of the “Estimates of the Common Relative Risk (Row1/Row2)” table produced by SAS Proc Freq. The adjusted Cochran-Mantel-Haenzel Chi-Square (CMH) statistic tests if a significant difference between Willits and ROC remains after controlling for cohort. The Breslow-Day Chi-Square (BD) test assesses if risk is consistent across cohorts. A P-value less than or equal to 0.05 rejects the null hypothesis. All programming was in SAS, including macros previously developed by us. Programs are available on request.

For our secondary analyses, we used other sources to evaluate demographic and access disparities, geographic stability, and air quality. The intent was to see if Willits was so different from ROC that those differences could explain outcome differences.

## Results

### Illness burden

We analyzed 796,917 diagnoses and 289,980 procedures found on 117,799 hospital admissions of 43,234 patients with SSN who were born between 1940 and 1980 who lived in the County at some time between 1991 and 2012. This population spent 521,116 days of their lives in hospitals, and hospital charges in this small county, unadjusted for inflation, totaled $3.6 billion. Of these patients, 7,564 lived in Willits.

Table 1 summarizes hospitalizations for men and women. The first comparison examines case rates based on unduplicated people. It shows Willits had a higher rate of cases per person-years than ROC and rates were unequal across cohorts for men (BD P-value = .0162) but not women. Specifically, among men, case risk concentrated in the 1950–1980 cohorts. Among both men and women, the 1980 cohort had the highest case risk. The overall adjusted number of excess cases attributable to living in Willits for men was estimated as 301 (CL 200–398) and 696 for women (CL 569–820).

**Table 1:** **Detailed statistics by sex for birth cohorts 1940–1980 over the period 1991-2012**

		Cohort rate per 10,000 person years					Excess cases			Adjusted relative risk			CMH Chi-square		BD Chi-square	
Measure	Area	1940	1950	1960	1970	1980	N	LCL	UCL	Ratio	LCL	UCL	Value	P-Val	Value	P-Val
**Male**																
Person	ROC	369	287	230	194	132										
	Willits	371	329	274	224	162	301	200	398	1.12	1.08	1.17	31.73	0.0000	12.16	0.0162
	RR	1.00	1.15	1.19	1.16	1.23										
Total discharges	ROC	1078	839	547	394	270										
	Willits	1161	970	685	524	313	1102	942	1258	1.16	1.14	1.19	160.3	0.0000	30.39	0.0000
	RR	1.08	1.16	1.25	1.33	1.16										
Total days	ROC	3912	3238	2329	1850	1367										
	Willits	3904	3541	2664	2585	1550	3974	3647	4297	1.11	1.10	1.12	506.5	0.0000	419.4	0.0000
	RR	1.00	1.09	1.14	1.40	1.13										
**Female**																
Person	ROC	368	332	456	560	318										
	Willits	427	383	519	651	397	696	569	820	1.17	1.13	1.21	100.8	0.0000	3.70	0.4486
	RR	1.16	1.16	1.14	1.16	1.25										
Total discharges	ROC	1064	876	1085	1295	694										
	Willits	1428	1052	1298	1639	917	2898	2898	3088	1.26	1.24	1.28	680.0	0.0000	29.20	0.0000
	RR	1.34	1.20	1.20	1.27	1.32										
Total days	ROC	3753	3071	3025	2992	1858										
	Willits	4559	3357	3413	3499	2198	7067	7067	7067	1.16	1.15	1.16	1317	0.0000	154.9	0.0000
	RR	1.21	1.09	1.13	1.17	1.18										

The second comparison is based on total discharges. In addition to more cases, Willits cases had more admissions. For both men and women, risk was elevated for all cohorts and cohort risk was unequal. Among men, the 1960–1970 cohorts had greater risk; among women, the 1940 and 1980 cohorts had greater risk. Overall, Willits men had 1,101 (CL 942–1258) excess discharges and Willits women had 2897 (CL 2704–3088) excess discharges.

The third comparison evaluates number of days lost to hospitalization. The 1940 cohort of Willits men was not at greater risk, and the 1970 cohort of men was at greatest risk. Among women, the 1950 Willits cohort had the relatively lowest risk and all cohorts of Willits women had greater risk than ROC women. For Willits residents, the excess number of days lost to illness so severe as to require hospitalization converts to about 30 life years, most during crucial child rearing periods.

Other comparisons evaluate illness by body systems (see Additional file 1). In this file, RR is greyed if the cohort difference is statistically significant in favour of the hypothesis that something about living in Willits increased poor health. Here the question is whether a diagnosis or procedure assigned to a given Level 1 CCS body system was ever found.

Adjusting for cohort, Willits men were at increased risk for all measures except genitourinary system diagnoses and procedures, and gender based procedures and cancers. The 1940 male cohort was at increased risk only for digestive system diagnoses and cancers. Among men, risk was elevated unequally across cohorts for seven of seventeen comparisons. The range of condition-specific risk for Willits men compared with ROC men by cohort was as follows: 1950, 1.16-1.32; 1960, 1.19-1.42; 1970, 1.23-1.70; 1980, 1.17-1.75.

Willits women were at increased risk for all measures and cohort risk was unequal for only one comparison. Elevated condition-specific risk by cohort for Willits women compared with ROC women was as follows: 1940, 1.12-1.45; 1950, 1.19-1.41; 1960, 1.09-1.61; 1970, 1.17-1.73; 1980, 1.16-1.73.

**Figure 2:**
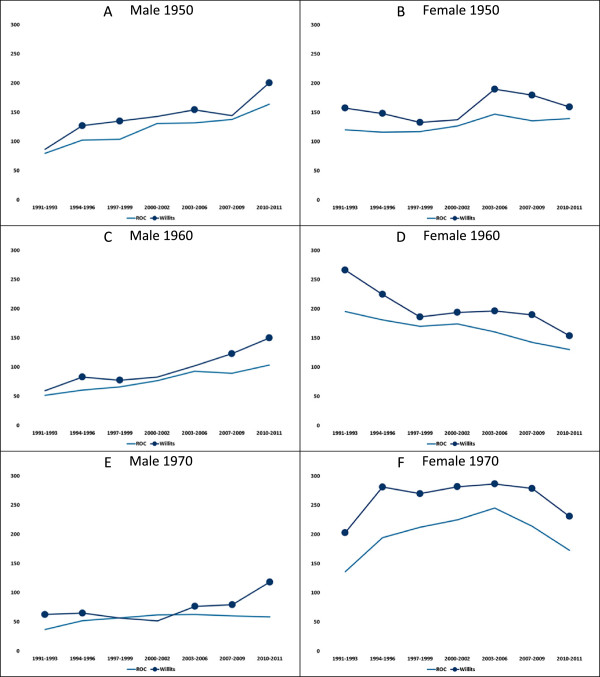
**Admissions per 10,000 person years by birth cohort, sex, and period.**

Figure 2 compares number of admissions by cohort over time. This shows how admission rates vary by cohort and time. The symbol is absent for periods when the Willits rate was not significantly different from the ROC rate. Among the 1940 cohort (see Additional file 1, tabs 1940M and 1940F), admission rates were little different for men but were consistently about 50% higher for Willits women. Admission rates for women in the 1980 cohort rose rapidly through their peak childbearing years (see Additional file 1, tab 1980F). Rates for this cohort of Willits women were more elevated in recent years, suggesting more issues than childbearing.

### Secondary analysis of demographic characteristics

**Figure 3:**
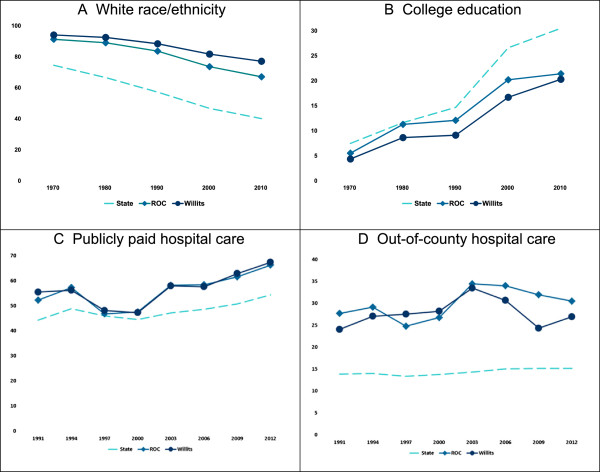
**Demographic and access disparities working age population 18–64 (%).**

We examined longitudinal demographic characteristics for the working age population [44, 45]. The County is disadvantaged demographically. It has a predominantly White (Figure 3A) population and a less educated work force (Figure 3B). During the 1990s, the County had an unemployment rate higher than the State, peaking in the years the Factory was closing [46].

This rural County has struggled with uneven health care access for its residents. The Federal government designated it medically underserved in 1991. It has three small hospitals with 155 beds. Outpatient clinics are at various locations including Willits. The County has persistent problems insuring its residents. By 2012, 54% of County adults age 18–64 were uninsured or publicly insured (public payer) [47]. In the PDD, County residents age 18–64 were about 25% more likely to have publicly paid care (Figure 3C) and about twice as likely to enter out-of-county hospitals than people living elsewhere (Figure 3D).

Population stability is an important community characteristic when chronic long-term exposure is considered. In terms of stability, Willits is similar to Love Canal, where 74% of families lived in the same house for most of the exposure period with a median exposure of 8.5 years [16]. The 1970–2000 Census [46] asked where families lived five years ago: in the same house, another house in the county, or outside the county. Willits was in the top quintile for percent of California residents living in the same house for five or more years with ROC close behind. More than 80% of Willits households lived in their current residence more than five years with 54% stable more than ten years. The Census no longer monitors long-term residential stability, so we are not able to update this. However, further supporting residential stability, a side analysis of the California Death Statistical Master file for the period 1991–2012 found more than 90 percent of decedents moved to the area before 1995, when the Factory closed, and 75% arrived before 1988 with about one year difference among women.

Willits is not substantively different from ROC. Both are disadvantaged relative to the State. The County is similar to communities labelled “back country” or “hard scrabble” in a study examining location of environmentally hazardous sites [48]. The authors estimated only military quarters had higher risk for being near hazardous sites than these types of communities. The County profile supports research that demographic inequities underlie the siting of environmentally hazardous facilities [48–51]. In the County, Willits had such a facility.

### Secondary analysis addressing air quality concerns

People opposed to the theory that this Factory caused ill health raise the issue of poor air quality so often that it must be considered a possible alternate explanation. County hospitals burned medical waste before California prohibited it. Backyard burning of rubbish was common before the County prohibited it. According to the 2010 Census, wood fuel heats 12.7% of County houses compared with 7% in Willits proper, 4.7% in the State and 1.7% nationally [44]. The County is heavily forested and has huge wildfires. It is in the heart of California’s “Golden Triangle” for growing marijuana, which Federal agents burn in the fields. Marijuana smoking is widespread. The County has a high rate of cigarette smokers [47], and is near the top among counties with the highest rates of need for substance abuse treatment [52].

In a side-analysis not reported here, we tested if higher illness in Willits may be due to higher rates of drug-related infections. To examine this, we calculated rates for all ten conditions under the CCS Infection/Parasitic group, which includes hepatitis, HIV, and sexually transmitted diseases associated with the drug culture. Rates for Willits men were not significantly different for any condition underlying that group. Willits women only had a higher rate of screening for infectious/parasitic conditions. This suggests physicians were trying to identify the underlying cause of their health problems.

Air quality reports from 1993 through 1997, when the Factory was winding down, indicate Willits had better air quality than other local communities [53]. More recent monitoring indicates air particulates have been low in the County and the Willits highway corridor [54].

Nonetheless, smoke is a known exposure route for Cr6 and other heavy metals. It occurs in high levels in wildfires, wood-burning stoves, and tobacco, methamphetamine, and marijuana smoke [9, 55–58]. California identifies marijuana smoke but not plants as a cancer-causing agent. Marijuana smoke contains 33 of the same harmful chemicals as tobacco smoke, including Cr6 [59]. Grant et al. found Cr6 in tobacco smoke ranged from 0.24-6.3 micrograms per gram, while marijuana smoke ranged from 5.9-16.3 [60]. They concluded heavy metal risks of marijuana smoke are no less than heavy metal risks of tobacco smoke.

Air-borne pollutants damage health, but are not particular to Willits in this County. Many residents smoke tobacco and marijuana. Available air quality statistics suggest local air quality is good. Thus, it is unlikely that air quality differences explain health outcome differences. Nonetheless, the presence in smoke of chemicals such as Cr6 and other heavy metals that are known carcinogens, known to impair the immune response, and implicated in respiratory and other chronic diseases is important public health information and is important for communication of risk related to exposure.

## Discussion

This is a retrospective public health surveillance study, defined as a systematic collection, analysis, and interpretation of data essential to plan, implement, and evaluate public health practice. It is retrospective because the analysis was as long as 22 years after physicians recorded the health information. It falls within the discipline of social epidemiology because it seeks to understand environmentally caused illness arising from public policy [45], specifically to permit building the Factory in Willits.

Using a cross-sequential design, we tested if Willits had an excess rate of adverse health conditions classified using standard federal definitions, when compared to people of the same sex and cohort in ROC. Admissions were during the last five years of Factory operations and 17 years thereafter. The principal diagnosis was of such severity that hospitalization was needed. We classified all diagnoses and procedures recorded upon discharge, then we summarized person-level classifications by sex, birth cohort, and area. To evaluate differences, we calculated relative risk by cohort and excess cases adjusted for cohort risk.

As is common for morbidity studies, women had more admissions and thus more diagnoses than men did. Not only did Willits residents have more admissions and days of care per population, they had more operating room procedures and women had more cancers, all indications of serious illness. With about 30 life years of excess hospitalization in this small community, the burden on families to care for children and children to care for parents while sick and hospitalized is incalculable. With about 60% of adults age 18–64 uninsured or publicly insured, the economic burden of these excess hospitalizations on the public is enormous.

Other health officers and researchers concluded exposure to contaminants was high when the Factory was operating [1]. The range of symptoms elevated among Willits residents is consistent with the complex effect of various toxics used there.

Disease was classified with reasonable accuracy. At discharge, physicians responsible for the patient identify the principal and up to 24 secondary diagnoses and 20 procedures summarizing illness. Coding rules specify secondary diagnoses be coded only if the physician considers them relevant to treating the principal condition for which the patient was admitted. Physicians identify these conditions during the course of care, and finalize their listing at discharge. Hospital records are less subject to recall bias because physicians use them to develop treatment plans and bill for care. To facilitate surveillance and outcome research, AHRQ developed the software we used to classify standard health conditions and procedures.

For decades, Willits and ROC have been demographically comparable and disadvantaged relative to the State. The County profile supports research that demographic inequities underlie siting of facilities associated with toxic hazards. With similar population disadvantages, stability, and air quality, siting the Factory in Willits versus elsewhere in ROC is a plausible explanation for health differences.

The design did not permit us to identify Factory employees or family members. People will be classified incorrectly if they attended school or worked in Willits (at the Factory or otherwise) and lived elsewhere. People are not in the study who lived in the County but never were hospitalized while living there, or who lived there and left before 1991, whether or not ever hospitalized. Although data suggest population stability, we do not know how long people lived in the County, when they arrived, or when (or if) they left. We have no way to assess or overcome these limitations.

When we began this work in 2007, we used population estimates available then. In updating our work, we used current Federal population data, and extrapolated ZIP-level Census estimates. These estimates may be biased. However, we have used three different estimates since beginning this work, with consistent results.Some researchers challenge ecological studies because they may lead to erroneously attributing illness at the group level to the individual. We clearly are studying group outcomes. We strongly urge a well-designed study to collect data about individuals who lived in Willits during childhood and the reproductive period. Figure 1 suggested they could be at heightened risk, and results are consistent with this possibility.

## Conclusions

The study was doable because many County patients had SSN and census data indicated a stable population. With these conditions met, the methodology is relatively simple. The results demonstrate the strength of the seldom-used cross-sequential design. By focusing on when people were born (cohort) rather than age, we have shown it is possible to use a longitudinal administrative dataset to evaluate long-term health outcomes. Our hope is that this report encourages population health researchers to consider this design in the future.

High admission rates generally reflect lack of access to preventive care, a sicker population, or both. The County is disadvantaged, with no substantive differences between Willits and ROC. Lower status communities tend to have poorer health. Communities with profiles similar to the County are more likely to host toxic sites, in this case Willits. Despite similar demographics, health access, residential stability, and air quality, Willits had more adverse health outcomes than ROC had.

There is little information on the long-term health effect for people exposed non-occupationally to Cr6 or related toxics during childhood or the reproductive period. Using a cohort-period (cross-sequential) design, we have shown it is possible to study the health of the Willits population. A followup study is needed, to focus on people born after 1950 who lived, worked, or attended school in Willits from 1963 forward.

## Authors’ information

LLR began to study Willits after a friend described the problem the residents faced. She became intrigued when she learned public health officials had opined it would be too difficult and expensive to do a study. TC agreed to help once he heard about Willits. After completing most of the first analysis, LLR contacted an attorney for a group of Willits residents suing the Factory and described the results. He asked her to be an expert witness and paid TC a modest amount for statistical consultation. The litigation ended several years ago.

## Electronic supplementary material

Additional file 1: **Statistics by sex summarizing illness for birth cohorts 1940-1980 over the period 1991-2012.** Description of data: tables for men and women with statistics on specific body system illness and when admissions occurred, with trend graphs. (ODS 125 KB)

## Abbreviations

AHRQ: Agency for Healthcare Research and Quality
ATSDR: Agency for Toxic Substance and Disease Registry
BD: Breslow-Day Chi-square test for homogeneity
CCS: Clinical Classification Software
CDHS: California Department of Health Services
CL: 95% confidence limit
CMH: Adjusted Cochran-Mantel-Haenzel Chi-Square test
Cr6: Hexavalent chromium
EC: Excess cases attributable to living in Willits
LCL: Lower 95% confidence limit
PDD: Patient discharge data
PHA: Public Health Assessment
ROC: Rest of County
RR: Relative risk
SSN: Social Security number, encrypted to protect confidentiality
UCL: Upper 95% confidence limit.
